# Clinical and Microbiological Evaluation of Diode Laser and Systemic Doxycycline as an Additive to Scaling and Root Planing for Stage II and Stage III Periodontitis Patients

**DOI:** 10.7759/cureus.56509

**Published:** 2024-03-19

**Authors:** Tapaswi A Kamble, Neeraj C Deshpande, Monali Shah, Rashmi Jha, Aayushi Shah

**Affiliations:** 1 Department of Periodontology, K. M. Shah Dental College and Hospital, Vadodara, IND

**Keywords:** root planing, scaling, diode laser, doxycycline, periodontitis

## Abstract

Aim: To assess and contrast the effectiveness of systemic doxycycline and diode laser as supplements to scaling and root planing (SRP) in terms of clinical and microbiological parameters.

Materials and methods: A total of 33 patients diagnosed with periodontitis stages II and III were included and randomized into group A (SRP + diode laser), group B (SRP + doxycycline), and group C (SRP alone). Selected sites were assessed for clinical and microbial parameters-plaque index (PI), gingival index (GI), pocket probing depth (PPD), relative attachment level (RAL), and colony-forming units (CFUs). Every clinical parameter was noted at baseline and after three months. The arithmetic mean, followed by the standard deviation, was calculated for the required assessment intervals. Analysis of variance (ANOVA) was used to compare all parameters between groups, and if the results of the ANOVA test were significant, post hoc analysis was performed. For intragroup comparison, student t-tests were performed.

Results: The clinical parameters significantly improved within three months for all groups. In terms of relative attachment level, a statistically significant difference (P < 0.001) was obtained at the three-month interval compared to the baseline value, with the most statistically significant difference seen in group A (3.36±0.50 to 0.64±0.50), followed by group B (3.18±0.40 to 2.18±0.40). The mean pocket probing depth observed at three months, compared to the baseline value, showed a statistically significant difference (P < 0.001) in group A (5.91±0.70 to 2.18±0.40) compared to group B (6.18±0.75 to 4.36±0.50), followed by group C (5.82±0.75 to 5.27±0.64).

Conclusion: The use of diode laser-assisted pocket disinfection and systemic doxycycline, in addition to scaling and root planing, has proven to be efficient for treating periodontal pockets.

## Introduction

Chronic inflammation is a symptom of periodontitis caused by dysbiosis in plaque biofilms, leading to the gradual degeneration of the structure-supporting teeth. It is characterized by the presence of periodontal bacteria, a conducive environment, and a vulnerable host. A vulnerable host refers to an individual who exhibits an inflammatory reaction to pathogens but lacks the ability to effectively combat the disease. The primary immunological factor causing the illness is the host’s harmful inflammatory response, while the primary microbial factor is a change in the composition of the oral microflora [1].

Dysbiosis, also known as microbial shift, refers to the phenomenon where certain diseases result from a decline in advantageous symbionts and an expansion of pathogenic organisms. In the context of periodontitis, it is widely accepted that the oral microbiota undergoes a transformation from predominantly gram-positive aerobes to predominantly gram-negative anaerobes. Gram-negative perio-pathogens like *Porphyromonas gingivalis* and *Aggregatibacter actinomycetemcomitans *display a significant positive association with chronic periodontitis, particularly *P. gingivalis*, which has been linked to the progression of periodontal disease. This bacteria, with a black pigment, produces a variety of virulence factors that directly or indirectly contribute to the destruction of periodontal tissues by influencing the host's inflammatory response [2].

The clinical response to periodontal therapy improved after eliminating this specific pathogen [3,4]. The primary goal of this therapy is to reduce and regulate bacterial colonies in both supragingival and subgingival areas. However, the inaccessibility of subgingival pathogenic bacteria may lead to treatment failure and the recurrence of periodontal inflammation [5,6]. Consequently, it is suggested that treatment strategies should be developed to specifically target and suppress the growth of certain periodontopathogens while controlling their proliferation on the tooth surface.

In addition to periodontal therapy without surgery, systemic administration of antimicrobial agents is employed. These agents exert their effects in the subgingival regions through the gingival crevicular fluid, effectively targeting areas that may be inadequately cleaned by mechanical instrumentation [7,8]. Tetracycline, currently under consideration for periodontal treatment, offers a broad spectrum of action. It acts as a bacteriostatic agent, inhibiting protein synthesis and effectively combating rapidly multiplying bacteria. Furthermore, it possesses an anti-collagenase effect, aiding in the prevention of tissue degradation and promoting bone resurgence. Doxycycline, a semi-synthetic derivative of tetracycline, exhibits enhanced compliance due to its complete absorption through the gastrointestinal tract [9].

Doxycycline functions as a bacteriostatic medication by reversibly binding to the 30S subunit of the ribosome in susceptible organisms, thereby inhibiting protein synthesis [10]. In the field of periodontics, laser technology has emerged as a captivating tool, with lasers such as CO_2_, Nd:YAG, Er:YAG, diode, and argon being extensively utilized as adjuncts in periodontal therapy [11]. The diode laser incorporates a solid-state semiconductor composed of aluminum, gallium, arsenide, and occasionally indium, enabling it to generate laser wavelengths spanning from 810 nm to 980 nm [12]. With its antibacterial characteristics, the diode laser system possesses photothermal properties that facilitate the simultaneous removal of granulation tissue and inflamed periodontal tissue (sulcular debridement). This process involves coagulation at a temperature of 60°C, leading to protein denaturation and a decrease in proinflammatory cytokines [12-14].

During the diseased periodontal pocket, the application of ablative diode laser photonic energy yields multiple advantageous effects. There is a decrease in the bacterial population of periodontitis-causing pathogens, such as *Aggregatibacter actinomycetemcomitans*, a part of the periodontal pocket-violet complex system, and *Porphyromonas gingivalis*, a component of the red complex system. This reduction occurs without causing harm to the underlying connective tissues, as these laser wavelengths can easily permeate the sulcular epithelium, resulting in a bactericidal effect. The targeted absorption of photonic energy by brown/black-pigmented anaerobic bacteria helps achieve the desired clinical outcome. Additionally, there is a debridement effect that aids in the removal of inflammatory substances. As a result of using the ablative diode laser, there is a notable improvement in periodontal attachment due to the reduction of inflammatory markers and a profound growth in cellular proliferation and lymphatic transmission, leading to a regenerative effect. Furthermore, the application of the diode laser provides effective relief from post-operative pain.

The use of low-energy laser light has demonstrated its effectiveness in reducing pain, expediting wound healing, and improving inflammatory processes. Specifically, irradiation with gallium arsenide (Ga-As) lasers has been observed to influence fibroblast proliferation. This proliferation of fibroblasts induced by laser treatment may be linked to the autocrine synthesis of growth factors. Moreover, low-power laser radiation can enhance DNA production, collagen, and procollagen levels [14], as well as promote the growth rate of cellular proliferation [15], and modify the locomotory characteristics of connective tissue cells. Additionally, exposure to an 830-nm GaAlAs laser inhibits prostaglandin E2 and IL-1b production, leading to gene expression that provides therapeutic benefits in the progression of gingivitis and periodontitis caused by microbes [16]. To the best of our knowledge, no research has been done to compare the effectiveness of applying a diode laser as an adjuvant to initial scaling and root planing (SRP) with administering systemic doxycycline. Thus, the purpose of this study was to evaluate the effects of diode laser and systemic doxycycline applied as adjunctives to scaling and root planing (SRP) on microbiology and clinical outcomes.

## Materials and methods

This clinical evaluation was conducted at the Department of Periodontology, and prior to its commencement, ethical clearance was obtained from the institution's ethical committee.

The study included patients in good physical health and oral hygiene, aged 18 to 60. Participants with stages II and III periodontitis, having a minimum of two teeth with a pocket probing depth exceeding 5 mm and a gingival index exceeding 2 mm, were selected for microbial testing and enrolled in the study. Exclusion criteria comprised individuals who had received antimicrobial and periodontal therapy within the previous six months, smokers or tobacco product users, pregnant or lactating mothers, and patients with known allergies to doxycycline. In total, 33 patients were included in the research.

**Figure 1:**
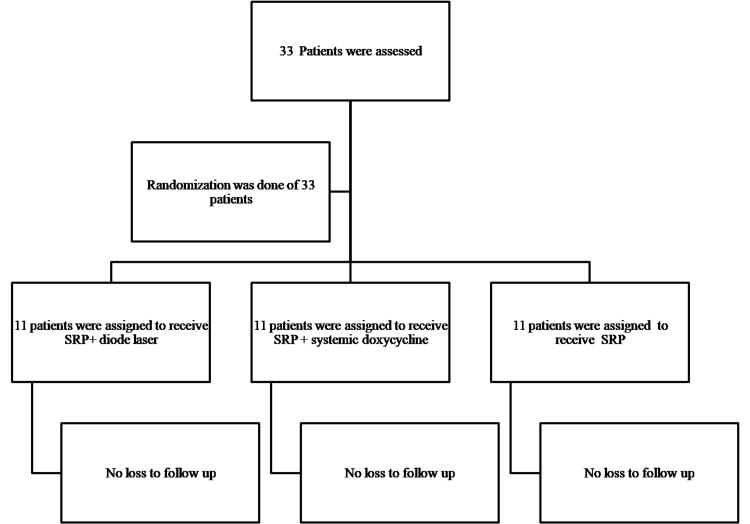
Participant flowchart. SRP: scaling and root planing.

Three groups were randomly assigned among eligible patients, each consisting of 11 participants, using a random allocation method performed by the primary investigator. The groups were designated as follows: group A received scaling and root planing (SRP) along with diode laser pocket disinfection, group B received SRP accompanied by systemic doxycycline, and group C received SRP alone. The collection of data, storage of collected data, and participant follow-up were managed by another investigator (Figure 1).

Plaque samples were collected from representative areas, and microbial analysis was conducted at baseline and after three months. Periodontal and microbiological measurements, including the plaque index, gingival index, pocket probing depth, relative attachment level, and colony-forming units (CFUs), were evaluated during both time periods. To standardize pre- and post-measurements, acrylic stents and University of North Carolina-15 probes were utilized.

Treatment Protocol

The initial periodontal therapy, SRP, was conducted in each treatment group at baseline. In group A, an additional pocket disinfection procedure was performed using a diode laser. The procedure involved directing the laser in an apico-coronal direction for one minute for each pocket, with the laser tip kept parallel at a 30-degree angle. Water irrigation was applied after SRP. In group B, an additional systemic dose of doxycycline (100 mg, once daily) was prescribed for seven days, and antibiotic administration commenced on the first day of periodontal therapy. In group C, only initial periodontal therapy, i.e., SRP, was performed without additional procedures.

Microbiological Analysis

A microbiological analysis was conducted to assess the colony-forming units (CFUs) of *Porphyromonas gingivalis* and *Aggregatibacter actinomycetemcomitans*. Intragroup comparisons were performed using two-tailed paired Student t-tests, while intergroup comparisons were subjected to analysis of variance (ANOVA).

## Results

Both groups exhibited indistinguishable baseline characteristics at the beginning of the trial, as depicted in Table 1.

**Table 1:** Comparison of all periodontal parameters in relation to the mean (SD) at baseline within the groups applying the ANOVA test. GI: gingival index, PI: plaque index, PD: probing depth, RAL: relative attachment levels, ANOVA: analysis of variance, SD: standard deviation.

Parameters	Group A	Group B	Group C	P-value
GI	2.77±0.19	2.78±0.17	2.69±0.29	0.59
PI	2.83±0.12	2.77±0.17	2.71±0.16	0.23
PD	5.91±0.70	6.18±0.75	5.82±0.75	0.49
RAL	3.36±0.50	3.18±0.40	3.18±0.40	0.54

After a duration of three months, significant improvements were observed in all assessed clinical outcomes for each of the three groups, except for the relative attachment level in group C (treated with SRP alone). Both group A and group B exhibited a statistically significant reduction in mean gingival index values from baseline to follow-up, with values changing from 2.77±0.19 to 0.20±0.06 and 2.78±0.17 to 1.30±0.20, respectively. However, group C did not demonstrate statistical significance, with a p-value of 0.037 (Table 2). In terms of the plaque index data, all three groups exhibited a statistically significant difference from baseline to the follow-up period, with a p-value of 0.001 (Table 2). The power of the study is 0.8.

**Table 2:** Intragroup comparison of gingival index and plaque index from baseline to three months using a paired t-test. *P<0.05-significant, GI: gingival index; PI: plaque index.

Parameters	Groups	Baseline	Three months	P-value
GI	Group 1	2.77±0.19	0.20±0.06	<0.001
	Group 2	2.78±0.17	1.30±0.20	<0.001
	Group 3	2.69±0.29	2.42±0.21	0.037
PI	Group 1	2.83±0.12	0.16±0.06	<0.001
	Group 2	2.77±0.17	1.55±0.21	<0.001
	Group 3	2.71±0.16	2.23±0.20	<0.001

Both group A and group B demonstrated a clinically significant reduction in pocket probing depth from baseline to the three-month follow-up, with values changing from 5.91±0.70 to 2.18±0.405 and from 6.18±0.75 to 4.36±0.50, respectively (p<0.001). In contrast, group C exhibited a decrease in pocket depth from baseline (5.82±0.75) to (5.27±0.64), with a p-value of 0.006 (Table 3).

**Table 3:** Intragroup comparison of probing depth, relative attachment levels, and colony-forming units from baseline. PD: probing depth, RAL: relative attachment levels, SD: standard deviation.

Parameters	Groups	Baseline (mean+SD)	Three months (mean+SD)	P-value
PD	Group 1	5.91±0.70	2.18±0.405	0.001
	Group 2	6.18±0.75	4.36±0.50	0.001
	Group 3	5.82±0.75	5.27±0.64	0.006
RAL	Group 1	3.36±0.50	0.64±.50	0.001
	Group 2	3.18±0.40	2.18±0.40	0.001
	Group 3	3.18±0.40	3.09±0.30	0.341
Colonies	Group 1	28.27±25.45	20.00±6.11	0.001
	Group 2	222.73±12.73	130.82±14.76	0.001
	Group 3	234.45±12.94	193.73±8.063	0.001

**Figure 2:**
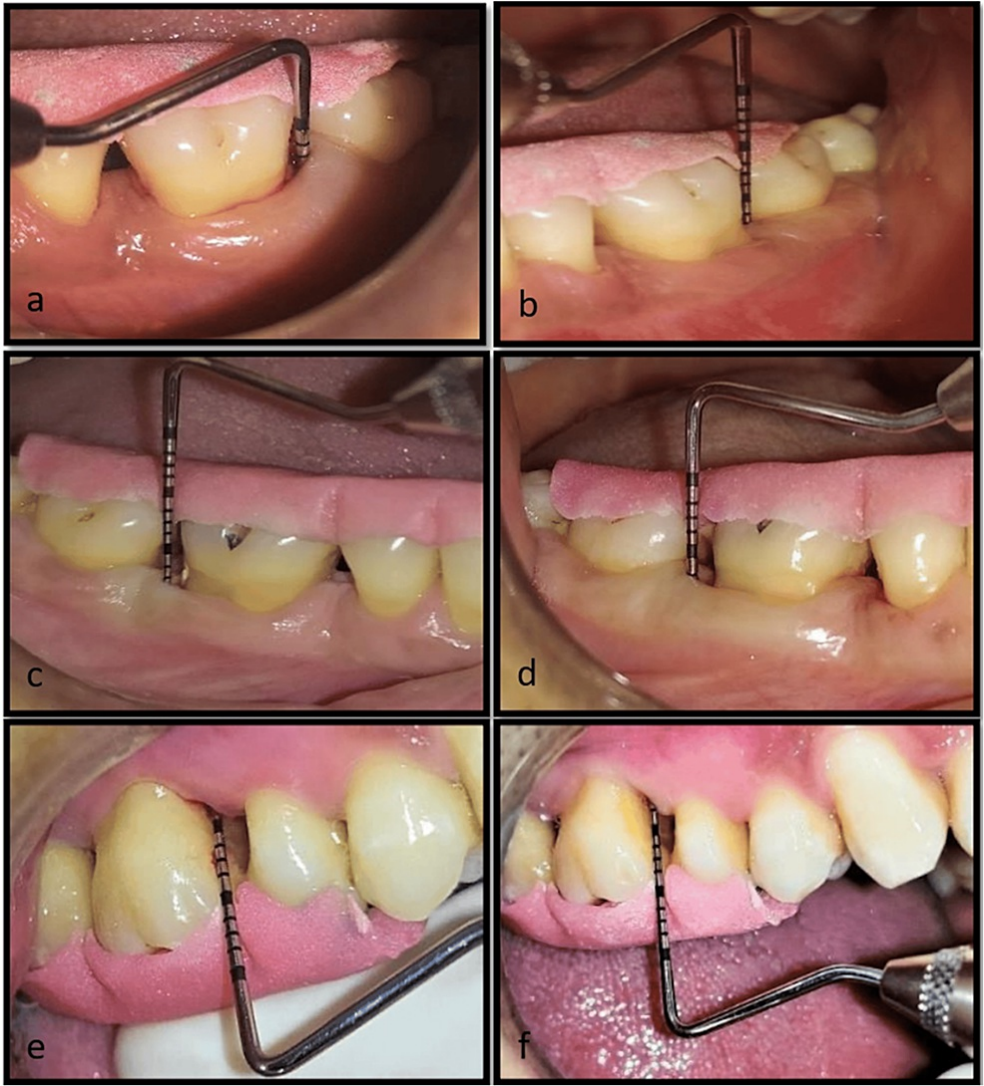
Group A: (a) RAL at baseline and (b) RAL at three months; group B: (c) RAL at baseline and (d) RAL at three months; group C: (e) RAL at baseline and (f) RAL at three months. Group A: Treated with SRP with diode laser; Group B: Treated with SRP with systemic doxycycline; and Group C: Treated with SRP alone. RAL: relative attachment levels; SRP: scaling and root planing.

Concerning the mean relative attachment level, both group A and group B demonstrated an increase from the initial value of 3.36±0.50 to 0.64±0.50 and from 3.18±0.40 to 2.18±0.40, respectively, at the three-month follow-up (p<0.001). However, in group C, there was a slight gain in attachment from baseline (3.18±0.40) to (3.09±0.30) at three months, which was not notably significant (p=0.341) (Table 3) (Figure 2).

All three groups exhibited a significant decrease in colony-forming units from baseline to three months, with a p-value <0.001 (Table 4).

**Table 4:** Intergroup comparison of the gingival index and plaque index, probing depth, relative attachment levels, and colony-forming units among all the groups using the ANOVA test. *p<0.05: significant, **p<0.001: highly significant. RAL: relative attachment levels; ANOVA: analysis of variance.

Variables	Group	Mean±SD	P-value
Gingival index at three months	Group 1	0.20±0.06	<0.001
	Group 2	1.30±0.20	
	Group 3	2.42±0.21	
Plaque index at three months	Group 1	0.16±0.06	<0.001
	Group 2	1.55±0.21	
	Group 3	2.23±0.20	
Pocket probing at three months	Group 1	2.18±0.40	<0.001
	Group 2	4.36±0.50	
	Group 3	5.27±0.64	
RAL at three months	Group 1	0.64±0.50	<0.001
	Group 2	2.18±0.40	
	Group 3	3.09±0.30	
Colony-forming units at three months	Group 1	20.0±6.11	<0.001
	Group 2	130.8±14.76	
	Group 3	193.7±8.063	

## Discussion

In the present study, the clinical and microbiological effects of diode laser and systemic doxycycline as adjuncts to SRP were investigated. At the three-month follow-up, statistically significant results were observed in all three groups in this randomized controlled trial. Upon comparing the groups, group A (diode laser with SRP) exhibited highly significant results compared to both group B and group C. Additionally, a statistically notable result was observed in group B when compared to group C. These findings align with the results reported by Akalin et al. [17] and Al-Nowaiser et al. [18], who also documented a significant decrease in gingival index scores following treatment with scaling and root planing, followed by systemic doxycycline administration during post-operative follow-up intervals.

The primary factors contributing to the decrease in depth following therapy are the gain in clinical attachment and the reduction of gingival inflammation, leading to the contraction of the pocket wall. The utilization of a laser with a low power level (1 W) on the tooth surface may be attributed to the photothermal effect, which helps in the elimination of periodontal bacteria from necrotic cementum and underlying dentinal tubules.

In this study, group A, which underwent both SRP and laser treatment (DL), exhibited a statistically significant average increase in relative attachment level from the preliminary measurement to the three-month period. These findings align with previous studies conducted by Üstün [19]. Treatment with all regimens demonstrated a notable reduction in pocket depth and an increase in clinical attachment. The primary reasons for the decrease in depth following therapy are the contraction of the pocket wall and the reduction in gingival inflammation. The CFU is a measurement used to determine the quantity of viable clonogenic cells per milliliter, indicating the number of cells that remain alive and capable of dividing to form small colonies.

In the present study, group A (SRP + DL) exhibited a statistically significant average decrease in the number of colony-forming units from baseline to the third month. This finding is consistent with previous research conducted by Singh et al. [20], Moritz et al. [21], and Kocak et al. [22], which also reported a significant decrease in CFUs. In the present study, group III (SRP alone) underwent microbiological investigation, revealing a statistically significant mean gradual decrease in colony-forming units from baseline to the third month. Euzebio Alves et al. [23] conducted a study that reported similar outcomes six months after conducting scaling and root planing. The gradual decline in colony-forming units could be attributed to the bactericidal action of the diode laser. The pigmented anaerobic periodontopathogens' protohemin and protoporphyrin pigments absorb the wavelength of diode lasers, leading to water evaporation and bacterial cell wall lysis, ultimately causing cell death. One limitation of the study is the relatively small sample size. Therefore, further investigation is necessary to comprehensively explore the potential of this laser technology, including extensive three-dimensional analysis and long-term follow-up studies.

## Conclusions

The study compared patients who underwent scaling and root planing with diode laser pocket sterilization to those who received scaling and root planing with the administration of systemic doxycycline and scaling and root planing alone. The group that received scaling and root planing in addition to diode laser treatment showed improved results compared to the other two groups. These supplementary benefits underscore the potential use of diode lasers as regular supplements to phase I therapy (SRP) for the treatment of periodontal pockets in subjects diagnosed with stage II and stage III periodontitis.
